# Perspective on the increasing role of optical wearables and remote patient monitoring in the COVID-19 era and beyond

**DOI:** 10.1117/1.JBO.25.10.102703

**Published:** 2020-10-21

**Authors:** Darren Roblyer

**Affiliations:** Boston University, Department of Biomedical Engineering, Boston, Massachusetts, United States

**Keywords:** wearables, remote patient monitoring, COVID-19, telehealth, portable

## Abstract

**Significance:** The COVID-19 pandemic is changing the landscape of healthcare delivery in many countries, with a new shift toward remote patient monitoring (RPM).

**Aim:** The goal of this perspective is to highlight the existing and future role of wearable and RPM optical technologies in an increasingly at-home healthcare and research environment.

**Approach:** First, the specific changes occurring during the COVID-19 pandemic in healthcare delivery, regulations, and technological innovations related to RPM technologies are reviewed. Then, a review of the current state and potential future impact of optical physiological monitoring in portable and wearable formats is outlined.

**Results:** New efforts from academia, industry, and regulatory agencies are advancing and encouraging at-home, portable, and wearable physiological monitors as a growing part of healthcare delivery. It is hoped that these shifts will assist with disease diagnosis, treatment, management, recovery, and rehabilitation with minimal in-person contact. Some of these trends are likely to persist for years to come. Optical technologies already account for a large portion of RPM platforms, with a good potential for future growth.

**Conclusions:** The biomedical optics community has a potentially large role to play in developing, testing, and commercializing new wearable and RPM technologies to meet the changing healthcare and research landscape in the COVID-19 era and beyond.

## 1 Introduction

**Fig. 1:**
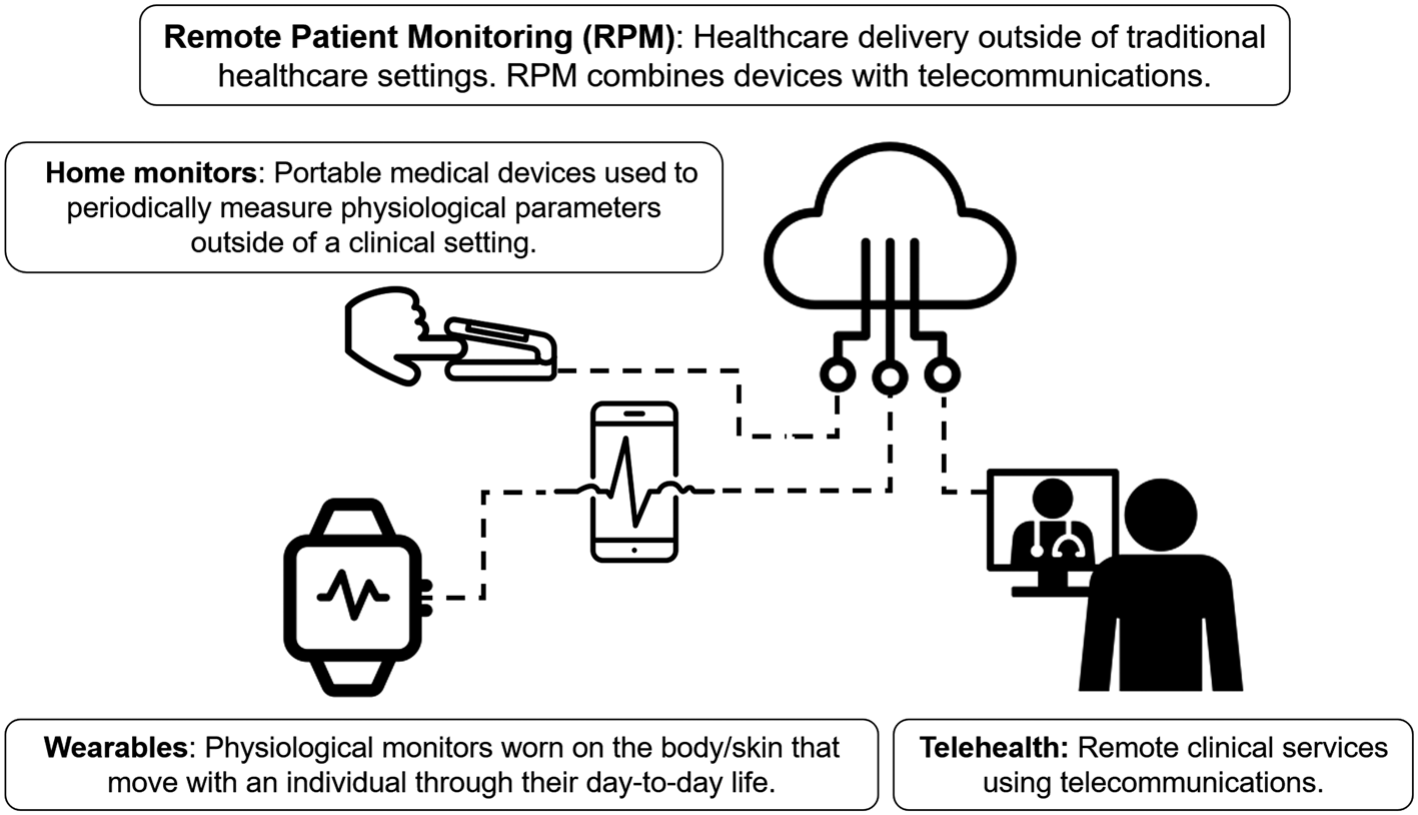
Wearables, home monitoring technologies, and telehealth combine to provide the framework for RPM. There has been a substantial increase in the implementation of telehealth and RPM technologies during the COVID-19 pandemic. There have also been regulatory changes to encourage further adoption of RPM technologies during the pandemic. This figure contains visual elements that are licensed under CC BY. (Visual elements of this figure, including finger, pulse oximeter, medical video visits, cloud, wearable device, and pulse, authored by iconsphere, IcoLabs BR, Bold Yellow US, Kiran Shastry IN, Shiva IN, and LAFS RU, respectively, are licensed under CC.)

Wearables are defined here as mobile physiological monitors worn on the body/skin that move with an individual through their day-to-day life. Home monitors are portable medical devices used to periodically measure physiological parameters outside of a clinical setting. Both wearables and home monitoring devices measure, analyze, and transmit health data.^1^ Remote patient monitoring (RPM) is a broad term that refers to the combination of medical devices, including wearables or portable home health monitors, with information technology solutions that allow health data to be communicated to a healthcare provider without in-person contact.^2^ RPM also includes telemedicine, in which a healthcare provider communicates and potentially tracks patient health using data streams from wearable or home monitoring technologies.^3^ Figure 1 shows how wearables and home monitoring technologies combine with telehealth to provide RPM.

Telehealth and RPM technologies were already playing a growing role in healthcare delivery prior to the COVID-19 pandemic.^4^ The COVID-19 pandemic and the governmental responses implemented to slow the spread of the disease have accelerated these trends across many countries and healthcare delivery systems. In this perspective, I first describe how recent research developments and regulatory changes have softened the ground for a shift in healthcare toward remote heath even prior to the pandemic. I then review how the COVID-19 pandemic has accelerated these changes. Finally, I review the current state of portable and wearable optical technologies and outline potential opportunities for the biomedical optics community to contribute to the shift toward RPM.

### 1.1 Use of Wearables and Adoption of Remote Patient Monitoring Were on the Rise Prior to the COVID-19 Pandemic

There are an increasing array of research and commercial home and wearable health monitors including, but not limited to, mobility trackers, blood pressure monitors, glucometers, digital stethoscopes, and electrocardiograms (ECG). The market for consumer wearables, which includes health trackers such as the Fitbit and Apple Watch, had revenues of almost $19 billion USD in 2019.^5^ Small, wrist-worn wearables track motion and heart rate using accelerometers and photoplethysmography (PPG), respectively. Newer versions can track additional parameters such as peripheral arterial oxygen saturation (${\mathrm{SpO}}_{2}$ or ${\mathrm{SaO}}_{2}$) using multiwavelength optical sensors, and electrical signals from the heart (ECG) using electrical impedance sensors. The adoption of these consumer wearables in the healthcare space is beginning to impact clinical study design and healthcare delivery. For example, academic and industrial research projects are now using data from these trackers to assess and predict a variety of health outcomes and disease states, including overall mortality, heart conditions, surgical recovery, mental health, and others.^6^^–^^8^ The well-publicized 2019 Apple Watch study, a collaboration between Stanford University and Apple Inc., monitored over 400,000 participants for signs of atrial fibrillation using an ECG monitor embedded in the watch.^9^ The Apple Watch study used a so-called “pragmatic” design, in which there were neither control nor intervention groups, but rather one cohort of participants who were tracked in their normal day-to-day lives. Participants showing signs of atrial fibrillation based on measurements taken by the Apple Watch were sent an at-home ECG patch to confirm the diagnosis. The study was “siteless,” and participants had no in-person interaction with study staff. Recruitment, consent, diagnosis, and follow-up were performed using a combination of a cellphone-based App, RPM technology (i.e., the Apple Watch), and telemedicine. These innovations provide a glimpse toward the potential future of RPM and wearable healthcare and research.

Healthcare providers are investing strongly in RPM and wearable technologies. The US market for RPM technologies is increasing at a compound annual growth rate of ∼20%.^10^ In 2019, a consulting group surveyed US healthcare providers and determined that 88% had invested or were evaluating investments in RPM technologies for chronically ill patients.^11^ This push in RPM investments was further encouraged by significant regulatory changes in the US occurring in 2018 and 2019. Until recently, it was challenging for healthcare providers to charge and receive reimbursement for RPM technologies and telehealth. Existing Current Procedural Terminology (CPT) codes, which are reimbursement codes used by the Centers for Medicare and Medicaid Services (CMS) for the US federal healthcare delivery programs, were limited for RPM technologies and telehealth prior to 2018. Since then, an older CPT code (99091) and four newer CPT codes (99453, 99454, 99457, and 99458) now allow for reimbursement of services that include training a patient how to set up and use RPM technologies, reviewing remotely collected RPM data, and consulting patients regarding their RPM data.^12^ These changes strongly suggest that regulators anticipated RPM technologies and telehealth as a growing part of healthcare delivery prior to the COVID-19 pandemic.

### 1.2 COVID-19 Pandemic Has Accelerated the Adoption of RPM, Wearables, and Telehealth

Stay-at-home orders and social-distancing rules implemented during the COVID-19 pandemic have accelerated the trend toward remote healthcare delivery in many countries. For example, in April 2020 during the peak of the initial wave of COVID-19 cases in the United States, telehealth claims accounted for 20% of submitted medical and dental claims in the northeastern US.^13^ This is compared with only 0.08% one year earlier.

The combination of telehealth with RPM technologies is likely to enhance the delivery of at-home healthcare. In response to the widely recognized need for improved at-home technologies that can assist in delivering healthcare during the pandemic, federal regulators have changed regulations or relaxed enforcement policies related to RPM technologies and telehealth. For example, CMS has allowed for equivalent reimbursements for in-person and telehealth appointments during the pandemic, helping to encourage the dramatic increase in telehealth.^14^ The US Food and Drug Administration (FDA) has changed some of their enforcement policies so that physiological monitors, including oximeters, spirometers, apnea monitors, ECGs, and others, which were previously cleared/approved for marketing to hospitals, can now be marketed toward in-home use without additional submissions.^15^ The relaxed regulations also allow hardware and software changes to be made to existing cleared devices to increase the ability of these devices to be used for RPM without seeking additional approval. The FDA has stated that these relaxed regulations will only remain in effect during the COVID-19 pandemic, but there are calls by the US administration and regulators for some of these changes to be made permanent.^16^

The COVID-19 pandemic has also led to changes in the way some clinical trials are conducted. Since many clinical trials had to reduce or stop enrollment during the pandemic,^17^ there has been a push by the National Institutes of Health (NIH) and National Cancer Institute (NCI) for technologies and telehealth to assist in monitoring patients taking part in clinical studies.^18^ So far these efforts have focused largely on remote consenting procedures and telehealth rather than adoption of new RPM technologies.^19^ However, even before the pandemic, the Clinical Trials Transformation Initiative (CTTI), a public–private partnership between the FDA and Duke University, called for a dramatic increase in mobile health technology use in clinical trials to improve the efficiency of clinical trials and reduce barriers while lowering costs. These mobile health technologies include RPM physiological monitors and wearables. The CTTI also advocates for “decentralized” clinical trial designs, which would rely heavily on RPM to recruit and monitor more diverse patient populations, including more women, rural citizens, and underrepresented racial groups.^20^

The pandemic’s impact on healthcare delivery will likely result in secondary health consequences that affect patients with many diseases, including chronic conditions such as cancer and heart failure. For example, the rate of cancer screenings has decreased, there have been delays in diagnosis and treatment, and the standard-of-care has been modified in some cases to accommodate patients during the pandemic. The consequences from these changes are likely to reverberate for years to come. The NCI estimates that there will likely be 10,000 excess deaths from breast and colon cancers over the next decade due to the impact of the pandemic on delayed screening and diagnosis.^21^ In addition, in breast cancer, it has been reported that more patients are undergoing neoadjuvant (presurgical) chemotherapy as surgeries are delayed.^21^ This change is aimed at reducing the amount of time patients are in the hospital, potentially reducing COVID-19 infection rates for these vulnerable populations. New optical RPM technologies may help address these secondary health consequences by increasing access to diagnosis, screening, and even treatment.

Wearable physiological monitors are also being tested for their potential to assist in COVID-19 diagnosis. Several universities and research institutes have partnered with consumer wearable companies including Fitbit, Garmin, and Apple to launch studies aimed at detecting early signs of COVID-19 infection. As with the Apple Watch study described earlier, some of these studies are using a pragmatic approach with passive monitoring of large study populations. One recently posted preprint analyzed 4642 smartwatch wearers who also had self-reported COVID-19 or other infections.^22^ The authors found that changes in heart rate, steps, and sleep were present in most COVID-19 cases, and that many of these changes were present prior to or at the onset of symptoms. They also reported an online, real-time detection algorithm that could identify 67% of cases at or before symptoms occurred. I refer readers to the recent review by Seshadri et al.^23^ for an in depth description of wearable technologies for COVID-19.

### 1.3 Areas of Opportunity for the Biomedical Optics Community to Meet the Needs of the Changing Healthcare Landscape

The changes described above provide substantial opportunities for the biomedical optics community to develop, test, and commercialize new optically based wearable and RPM technologies to meet the needs of changing healthcare delivery modes. There are already examples of increased optical RPM adoption during the pandemic. Pulse-oximetry, one of the most ubiquitous physiological measurements in healthcare, has undergone increased at-home use as reports indicate that low ${\mathrm{SpO}}_{2}$ may be an important indicator of COVID-19 infection.^24^ One recent study found that ${\mathrm{SpO}}_{2}$ levels <92%, when measured at home after an initial nonsevere COVID-19 diagnosis, was an indicator for the need for subsequent hospitalization.^25^ I note that these studies had small sample sizes, and thus results need to be rigorously confirmed.

Current commercial wearables and RPM technologies only utilize a small portion of the physiological data accessible with optical techniques. Fortunately, there has been a growing interest in academia and industry to develop more advanced and new optical wearables for RPM technologies. Table 1 contains an abbreviated summary of optically derived physiologic and diagnostic parameters relevant to wearables and RPM technologies. The table also lists examples of wearable or portable versions of optical techniques capable of measuring these parameters. The table is limited to *in vivo* physiological monitoring and does not include optical technologies for quantifying biosamples such as blood, urine, feces, etc. Examples include diffuse optical wearables for fitness monitoring,^30^ ambulatory monitoring,^31^ breast cancer therapy monitoring,^32^ and neuroapplications.^33^ There has also been substantial development of smartphone-based optical platforms for dermoscopy and point-of-care diagnoses for an array of diseases and conditions.^48^ Some of the technologies in Table 1, including low-cost colposcopy, capillaroscopy, and microendoscopy, have been developed for low-resource and global health applications.^29^^,^^49^^–^^51^ Overall, the optical technologies listed in Table 1 are well positioned to be adapted and implemented for RPM.

**Table 1:** Noninvasive optically derived parameters relevant to RPM currently available using wearable or portable platforms.

Optically derived parameter	Wearable or portable optical technique
Heart rate, HRV	PPG, LSI^26^
${\mathrm{SpO}}_{2}$	Pulse oximetry, porphyrin-based sensors^27^
Blood flow	SPG,^26^ SCOS,^28^ capillaroscopy^29^
Δ[Hb], ${\mathrm{StO}}_{2}$, ${\mathrm{SmO}}_{2}$	CW-NIRS,^30^^–^^34^ DRS,^35^ PA^36^
[Hb/Mb]	FD-DOS,^37^^,^^38^ TD-DOS,^39^ SFDI^40^
Optical scattering	FD-DOS, TD-DOS, SFDI,^40^ OCT,^41^ ESS
Glucose	Spectroscopy^42^^–^^44^
Blood pressure	PPG^45^^,^^46^
Images of the eye, skin	Ophthalmology,^47^ OCT,^41^ smartphone-based dermoscopy^48^
Images of the cervix	Colposcopy^49^
Images of the oral mucosa	Endogenous imaging,^50^ microendoscopy^51^

Note: HRV, heart rate variability; ${\mathrm{SpO}}_{2}$, peripheral oxygen saturation; [Hb], hemoglobin concentration; [Mb], myoglobin concentration; ${\mathrm{StO}}_{2}$, tissue oxygen saturation; ${\mathrm{SmO}}_{2}$, muscle oxygen saturation; PPG, photoplethysmography; LSI, laser speckle imaging; SPG, speckle plethysmography; SCOS, speckle contrast optical spectroscopy; CW-NIRS, continuous-wave near-infrared spectroscopy; DRS, diffuse reflectance spectroscopy; SFDI, spatial frequency-domain imaging; PA, photoacoustics; FD-DOS, frequency-domain diffuse optical spectroscopy; TD-DOS, time-domain diffuse optical spectroscopy; ESS, elastic scattering spectroscopy; OCT, optical coherence tomography.

While some of the parameters and associated technologies in Table 1 are already available in highly wearable or portable formats, including heart rate, PPG, and some types of tissue oxygen saturation measurements, other parameters and technologies will require substantially more development to become home or wearable technologies that can operate without expert users present. These include, but are not limited to, frequency-domain and time-domain diffuse optical spectroscopy (FD-DOS and TD-DOS), which can measure absolute concentrations of tissue chromophores including oxy- and deoxyhemoglobin, as well as optical coherence tomography (OCT), which can provide high-resolution imaging of tissues up to several mm’s deep. Recent advances in miniaturization of FD-DOS and TD-DOS detectors and electronics, and portable OCT technologies, suggest that highly portable versions of these techniques may be available in the future.^37^^–^^39^^,^^41^ These and similar technologies may be well suited to mobile-health clinics, outpatient clinics, and other so-called “off-site” settings in which some in-person contact is required. While not the focus of this perspective, these venues serve an important and growing role in healthcare, and the COVID-19 pandemic has increased their usage for applications such as wound care.^52^

Other considerations such as device cost, durability, and optical safety are highly important as optical wearables and RPM technologies will be used by nonexperts. Advances in computational imaging, compressive imaging, and deep learning are reducing hardware requirements and costs for cellphone-based and other optical imaging techniques.^53^ In addition, the ability of optical devices to utilize telecommunications to securely transmit health data to providers is essential for RPM implementation. Specific communications protocols between devices, smartphones, the cloud, and health care providers are of the utmost importance in terms of privacy, patient safety, and efficacy of healthcare delivery. For example, there has been a recent interest in using blockchain to provide a secure and decentralized means of sharing patient data.^54^ Finally, optical wearables and RPM technologies must synergize with telehealth technologies, and physicians need to be able to access and understand collected data sets easily and rapidly. This requires robust algorithms that analyze complex longitudinal data sets and present the health data in an accessible and familiar format to physicians, hopefully facilitating rapid adoption.

New optical wearables and RPM technologies have the potential to address many clinical scenarios related to the COVID-19 pandemic. Table 2 contains a brief summary of opportunities for remote and wearable optical technology development, both for COVID-19 and for the many secondary health consequences of COVID-19 including reduced screenings and delayed diagnoses, treatments, and rehabilitations.

**Table 2:** Opportunities for optics-based RPM during the COVID-19 era.

Target area	Need(s)
**COVID-19**	
Detection and diagnosis	Earlier at-home diagnosis, assessment of infection severity
Recovery	Remote vital signs during at-home recovery
**Secondary health effects related to the COVID-19 pandemic**	
Screening and diagnosis	Remote screenings and diagnoses for acute and chronic diseases
Treatment monitoring	Remote tracking of the effectiveness of medications, therapies, and other interventions
Surgical recovery	Remote vital signs during at-home recovery
Wound healing	Remote monitoring of healing, infection, necrosis
Clinical trial monitoring	Remote vital signs to determine side effects, toxicities, and efficacy of investigational drugs/interventions
Neuromonitoring	Remote monitoring of brain activity during rehabilitation and for basic science studies

## 2 Conclusion

There are substantial opportunities for the biomedical optics community to help address the myriad challenges associated with increased remote healthcare delivery in the COVID-19 era and foreseeable future. Investment and regulatory changes are helping to incentivize new technology development in RPM. The continued development and miniaturization of new optical wearables and RPM technologies that can be used by nonexperts remains a challenging goal for our community but with potentially dramatic rewards for safe and remote disease screening, diagnosis, treatment monitoring, rehabilitation, and treatment.
